# Mycosis fungoides with Psoriasiform plaques: A case report and review of the literature

**DOI:** 10.1002/ccr3.6848

**Published:** 2023-02-23

**Authors:** Amir Mohammad Beyzaee, Neda Jahantigh, Mohammad Goldust, Ghasem Rahmatpour Rokni, Mahsa Babaei, Bahare Ghoreishi, Sepideh Sadathosseini

**Affiliations:** ^1^ Department of Dermatology Mazandaran University of Medical Sciences Sari Iran; ^2^ Mazandaran University of Medical Sciences Sari Iran; ^3^ Department of Dermatology University Medical Center Mainz Mainz Germany; ^4^ Department of Dermatology, Faculty of Medicine Mazandaran University of Medical Sciences Sari Iran; ^5^ Shahid Beheshti University of Medical Sciences Tehran Iran

**Keywords:** biopsy, diagnosis, mycosis Fungoids, Psoriasiform plaques, psoriasis

## Abstract

Mycosis fungoides (MF) is the most common variant of primary skin T‐cell lymphoma. It typically manifests as an indolent progressing cutaneous eruption with erythematous scaly patches or plaques. Due to the nonspecific pathological findings, it can be easily misdiagnosed as psoriasis. A 34‐year‐old woman with a history of psoriasiform plaques for 12 years was referred to our dermatology clinic. In the beginning, the diagnosis of psoriasis was made and topical steroids were prescribed: it did not exhibit any clinical improvement. During the visit, skin biopsy was performed and the diagnosis of MF was confirmed. Treatment with PUVA, prednisolon, methotrexate, topical ointment including ucerin, urea, and clobetasol were initiated. Significant improvement in all lesions were observed after 1 month of the treatment, and within a year, the disease improved dramatically after PUVA therapy. In refractory cases of psoriasiform plaques that are progressive and/or ulcerative despite the optimal treatment, biopsy is required and a possible diagnosis of MF should be kept in mind.

## 1 INTRODUCTION

Mycosis fungoides (MF), a primary cutaneous lymphoma, is considered as the most common variant of skin T‐cell lymphoma with a prevalence of 50% among all types of skin T‐cell lymphoma. It mostly affects middle aged and elderly adults of all races.^1^ MF typically manifests as an indolent progressing cutaneous eruption with erythematous scaly patches or plaques and may progress to a generalized erythroderma and/or cutaneous tumors, or invade the extra‐cutaneous tissues. In the early stages, it usually mimics other common skin disorders including psoriasis, eczema, or cutaneous malignancies like tenosynovial sarcomas and dermatofibrosarcomas.^2^, ^3^, ^4^ The diagnosis of MF is challenging due to the nonspecific clinical and pathological findings, particularly at the early stages, making it highly possible to be misdiagnosed for years.^5^ A certain diagnosis is made by pathological findings of skin biopsy.^5^

Several therapeutic regimens with desirable reported outcomes^5^ are available for treating MF; topical treatments (topical corticosteroids, phototherapy, topical chemotherapy, topical retinoids and radiotherapy) for the early stages and systemic therapy (interferon‐α, oral retinoids like bexarotene, acitretin, histone deacetylase inhibitors, fusion toxin denileukin diftitox, and chemotherapy drugs) for advanced stages. The prognosis of the disease is highly depended on the type and extent of skin involvement, and extra‐cutaneous invasion.^1^, ^6^

Psoriasis is one of the most prevalent chronic inflammatory disorders of the skin with an auto‐immune origin. It mostly manifests with erythematous scaly plaques which are clinically similar to psoriasiform MF. Diagnosis of psoriasis is usually made clinically and treatment options are mainly consisted of immunosuppressive and biologic agents.^7^, ^8^

In this article, we report an uncommon presentation of psoriasiform MF in a patient who was misdiagnosed with psoriasis and was managed accordingly for a long time. We also review the latest articles concerning the diagnostic and therapeutic challenges of MF and its clinical‐pathological findings.

## 2 CASE PRESENTATION

**FIGURE 1:**
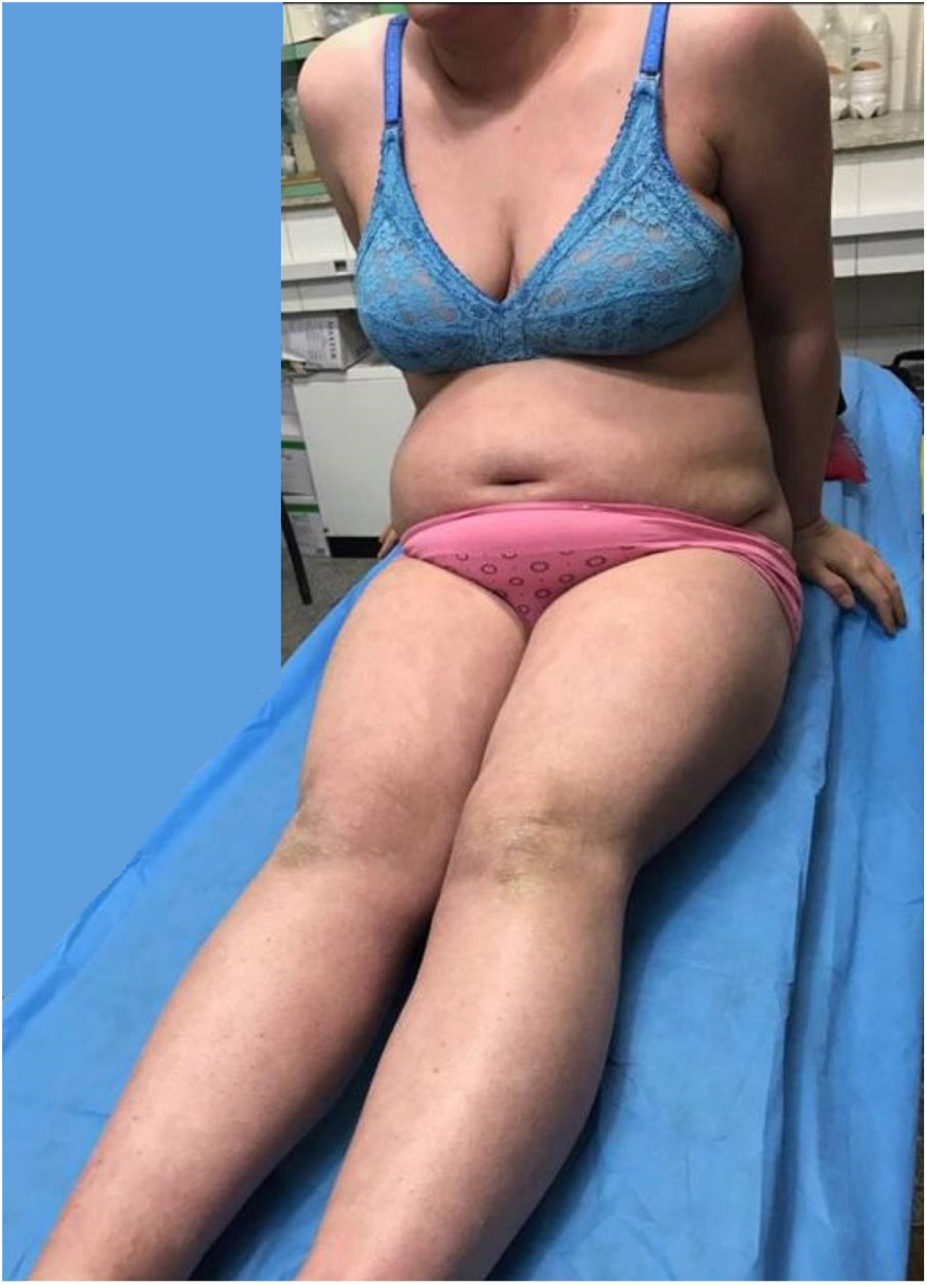
Before treatment: presence of indurated, erythematous plaques with thick psoriasiform scales

**FIGURE 2:**
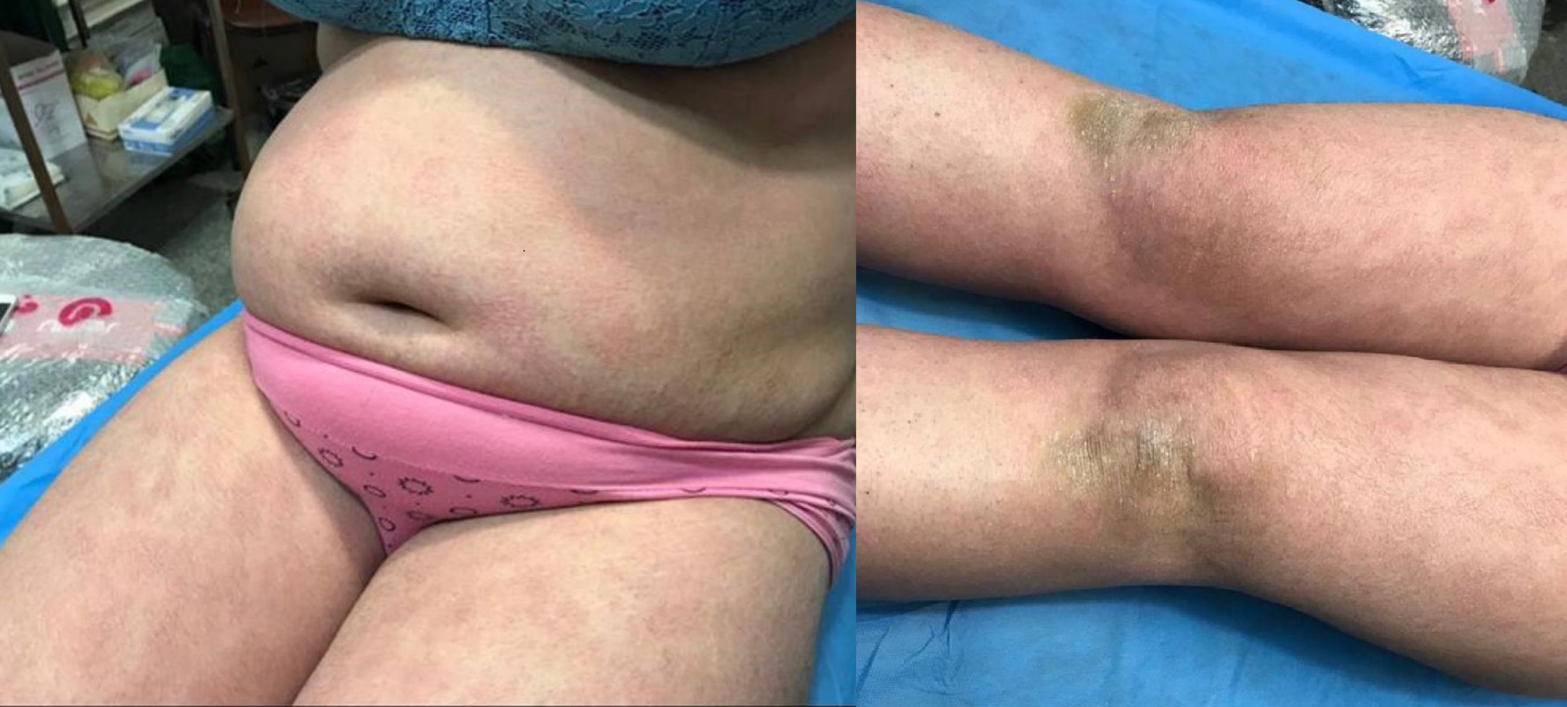
Before treatment: presence of indurated, erythematous plaques with thick psoriasiform scales

A 34‐year‐old woman was referred to our dermatology clinic complaining from psoriasiform plaques (Figures 1 and 2). The plaques appeared 12 years ago in multiple areas of the body and progressed gradually. No associating signs had been reported. Past medical history, pharmacological history, and family history were clear. During this time, the patient had been treated with topical steroids intermittently, with the diagnosis of psoriasis. After experiencing significant unsatisfactory results, the patient was referred to our dermatology department.

On examination, the patient was found to have multiple indurated, well‐demarcated erythematous plaques with thick psoriasiform scales all over her chest, abdomen, back, and upper and lower extremities. Her face was nearly spared. Her hair and nails were normal. No lymphadenopathy nor other systemic symptoms were found. Except for the cutaneous findings, physical examination revealed normal findings of other organs.

The lesions on the affected skin area were biopsied and the pathological report confirmed the diagnosis of MF at the presence of 60% neutrophils, 30% lymphocytes (3% atypical), 8% monocytes, and 1% eosinophils.

**FIGURE 3:**
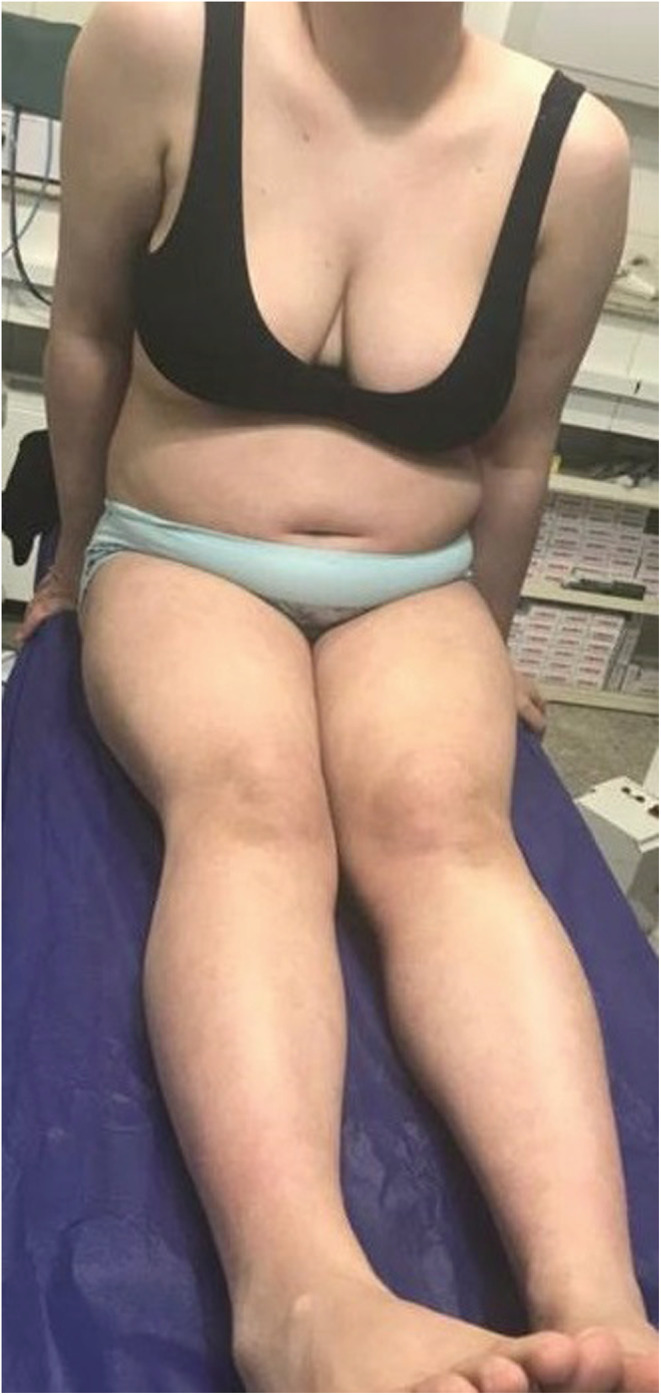
After treatment: improvement of lesions

**FIGURE 4:**
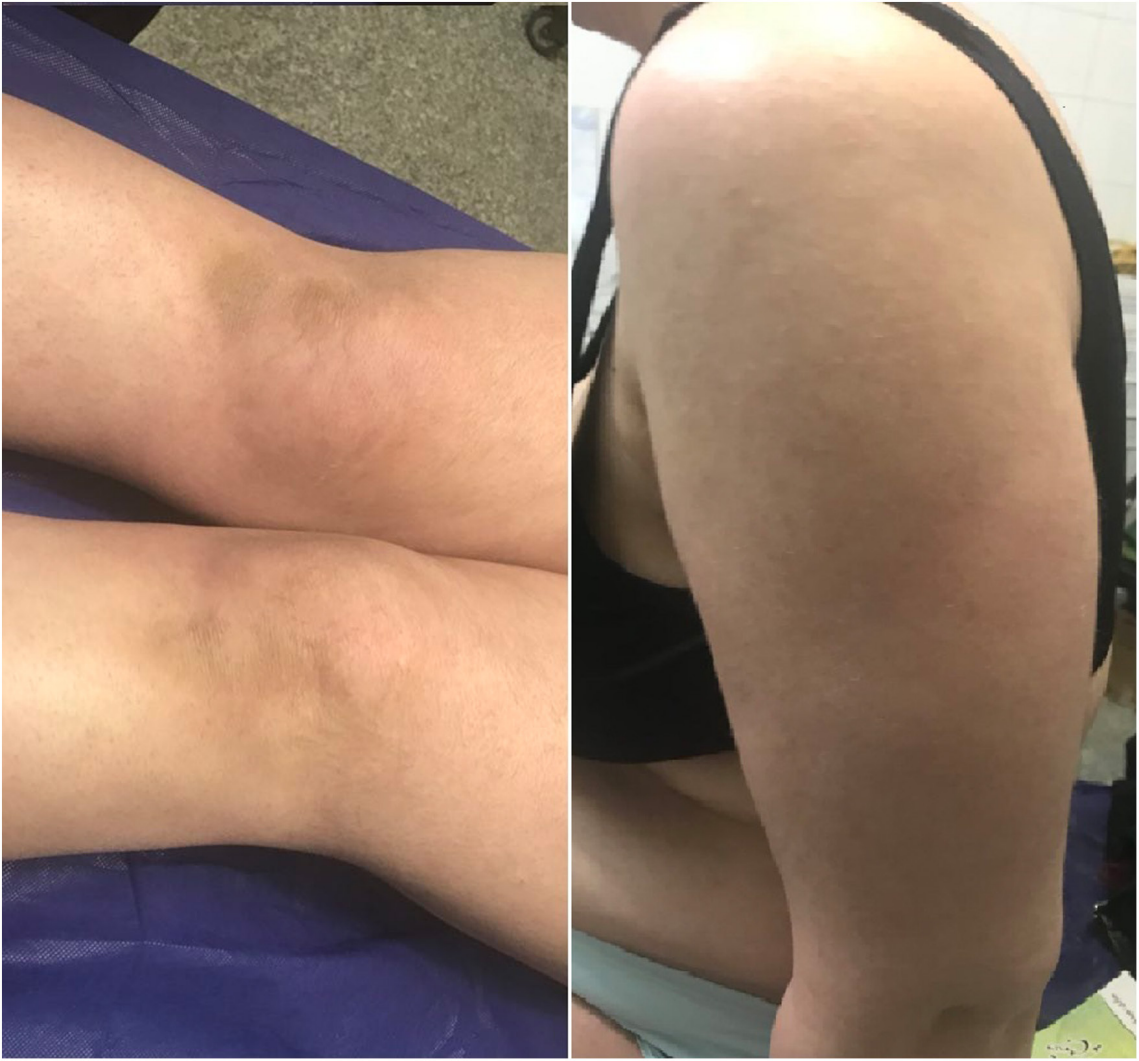
After treatment: improvement of lesions

The patient underwent PUVA therapy and weekly methotrexate injection; also, oral prednisolone 2.5 mg daily for the first month followed by 1.25 mg daily during the next months, a topical ointment including 90% ucerin and 10% urea once daily, and a topical ointment of 50% clobetasol and 50% ucerin once every night were prescribed. After 1 month of the treatment, significant improvement in all the lesions was observed. After 1 year of the treatment, the patient had completely recovered from the disease, and it was found that the treatment with PUVA was significantly efficient (Figures 3 and 4).

## 3 DISCUSSION

Mycosis fungoides shares similar manifestation and overlapping pathophysiological features with psoriasis. It is crucial to diagnose MF properly and differentiate it from psoriasis, because the systemic treatments such as cyclosporine, which is a highly effective treatment for psoriasis patients, significantly worsens the course of MF.^9^, ^10^

In most patients suffering from psoriasiform plaques, pathological findings are not supportive enough for psoriasis diagnosis, while nonspecific histopathological findings of MF in the early stages may be misdiagnosed as psoriasis.^11^ Similarly, our case was either a case of MF that was misdiagnosed as psoriasis and was prescribed with long‐term immunosuppressive treatments. This case, besides several cases that have been reported earlier, highlights the importance of consideration of MF, especially in patients with psoriasiform plaques that are not responsive to psoriasis treatment, and refractory cases. In these cases, skin biopsy is recommended as soon as possible, particularly before administration of biologic agents. Refractory cases of psoriasiform plaques with poor therapeutic response are required to receive thorough examination for MF using immunochemistry and molecular biology techniques, as well.^11^ Interestingly, combination therapy has shown satisfactory efficacy in cases of concomitant MF and psoriasis, and it can be considered when the diagnosis is not confidently confirmed.^11^ In this case, after histopathological confirmation of MF, combination therapy was initiated. Topical treatment consisted of two mixture ointments including 90% ucerin and 10% urea once daily, and another with 50% clobetasol and 50% ucerin once every night and systemic treatment included weekly methotrexate injection and oral prednisolone. PUVA therapy was also initiated with high efficacy for MF. Our case also shows the significant efficacy of PUVA besides combination therapy for the complete resolution of MF lesions and the underlying systemic inflammation. Previous studies highly recommend PUVA therapy in MF cases as soon as a diagnosis is confirmed. In refractory cases, low‐dose methotrexate is also highly recommended and a combination of topical medications is often required. A retrospective study investigated the occurrence and time of relapse in patients with early MF treated with narrowband UVB phototherapy. Of 31 patients, who were followed‐up for a mean of 56.5 ± 30.2 months (median 55 months, range 20–120 months), relapse was observed in 11 (35.5%) patients, within a mean of 28.8 ± 18.2 months (median 33 months, range 4–59 months), whereas 20 (64.5%) patients stayed relapse‐free for a mean of 54.2 ± 28.8 months (median 55.5 months, range 20–119 months). Patients received maintenance phototherapy with a median duration of 12 months (range 1–30 months) after achieving complete response.^12^ Results indicate that narrow band UVB phototherapy may induce low relapse rates and long relapse‐free intervals for early MF. Another retrospective study aimed to review the response rate of methotrexate in the treatment of MF. Sixty‐nine MF patients with patch/plaque and tumor stage, with a follow‐up period of 201 months were studied. The greatest number of patients (60) had patch/plaque stage T2 disease (≥10% skin involved). Of these, seven (12%) achieved complete remission and 13 (22%) achieved partial remission, with a total response rate of 20 out of 60 (33%). The median time of treatment failure was 15 months. Side effects caused treatment failure in six out of 69 patients (9%). Accordingly, low‐dose methotrexate may be valuable for treating a subset of MF patients with patch/plaque, who are resistant to other therapies.^13^ Moreover, a large prospective nonrandomized study investigated the efficacy of mechlorethamine hydrochloride and topical betamethasone in the treatment of early‐stage MF patients. Sixty‐four consecutive patients with newly diagnosed early‐stage MF (stage IA, n = 33; stage IB, *n* = 26; stage IIA, n = 5) were enrolled in the study. Patients were treated with an application of 0.02% aqueous solution of mechlorethamine followed by an application of betamethasone cream, twice a week for 6 months. They concluded that topical anti‐inflammatory agents alone are highly effective treatment for early‐stage of MF.^14^

## 4 CONCLUSION

Special attention is required for clinical cases of psoriasiform MF. In probable cases of psoriasis that are unresponsive to conventional treatment, progressive, or ulcerative, the need to perform a biopsy is indicated. Atypical cases also require skin biopsy before the initiation of immunosuppressive agents to rule out the possible MF. Moreover, MF is highly responsive to PUVA and upon confirmation of a diagnosis, the initiation of MF combination therapy is required.

## AUTHOR CONTRIBUTIONS

**Amir Mohammad Beyzaee:** Conceptualization; resources. **Neda Jahantigh:** Data curation; software. **Mohammad Goldust:** Methodology; writing – review and editing. **Ghasem Rahmatpour Rokni:** Project administration; supervision; visualization; writing – review and editing. **Mahsa Babaei:** Conceptualization; writing – review and editing. **Bahare Ghoreishi:** Investigation; software. **Sepideh Sadathosseini:** Investigation; visualization.

## FUNDING INFORMATION

None of the authors have received any funding for this study.

## CONFLICT OF INTEREST

All authors declare that they have no conflict of interests in this study.

## CONSENT

Written informed consent was obtained from the patient for publication of this case report and accompanying images.

## Data Availability

Data are available with the corresponding author upon request.
